# 

**Published:** 1902-12-06

**Authors:** 


### NOTICE TO CORRESPONDENTS.

All MSS., Letters, Books for Review, and other matters intended for the Editor should be addressed The Editor, "The Hospital," The Hospital Building, 28 & 29 Southampton Street, Strand, W.C.

The Editor cannot undertake to return rejected MS., even when accompanied by stamped directed envelope.

**Notice to Advertisers and Subscribers.**

All Advertisements, Orders for Copies of The Hospital, and Business Communications should be addressed to **THE MANAGER, (Not to the Editor)**, The Hospital, 28 & 29 Southampton Street, Strand, London, W.C.

The Cost of Subscription, post free, is as follows.—Per week, 2½d.; per quarter, 2s. 8d.; per half-year, 5s. 3d.; per annum, 10s. 6d. Foreign:—Per annum, 15s. 2d., payable, in all cases, in advance.

**NOTICE.—Vol. XXXII. of "The Hospital" (April to September 1902) in handsome cloth binding is now on sale at the office of this Journal, price, 7s. 6d. Cases for binding the Numbers contained in this Volume 1s. 6d. Index to Vol. XXXII., 2½d., post free.**

**The Monthly Part for December is now ready. Price 6d., by post 8d.**

### BOOKS RECEIVED.

W. and R. Chambers.

"Organic Chemistry." By W. H. Perkin, Junr., Ph.D., F.R.S., and F. Stanley Kipping, Ph.D., D.Sc. (Lond.), F.R.S.

Allman and Son, Ltd.

"Essentials of Domestic Hygiene." By Clare Goslett.

H. Kempton.

"The Science and Art of Prescribing." By Colbeck and Chaplin.

### The Student of Medicine.

### Appointments.

J. Acomb, M.R.C.S., L.R.C.P., appointed Resident Medical Officer at the Ida Semi-convalescent Hospital of the General Infirmary, Leeds. **John M. Ahern**, M.B., B.Ch., B.A.O.R.U.I., L.R.C.P.&S.I., appointed Deputy Medical Officer to H.M. Prison, Liverpool. **T. W. Bailey**, M.R.C.S.. L.R.C.P.Lond., appointed District Medical Officer of the Bromley Union. **A. P. Beddard**, M.D.Cantab., M.R.C.P., appointed Assistant Physician to Guy's Hospital. **Sidney Bree**, M.R.C.S., L.R.C.P.Lond., appointed Certifying Factory Surgeon for the Brantham District of the county of Suffolk. **Alec. H. Brewer, jun.**, M.R.C.S.Eng., L.R.C.P.Lond., appointed Honorary Anæsthetist to the Male Lock Hospital, Dean Street, Soho. **W. F. Chrispin**, L.S.A., appointed Medical Officer of Health to the Castleford Urban District Council. **Miss Alice M. Corthorn**, M.B., B.C.Lond., appointed Senior Assistant to the Out-patients at the New Hospital for Women. **J. S. Colquhoun**, L.R.C.P., L.R.C.S.Edin., appointed District Medical Officer of the Darlington Union. **C. de L. Carey**, B.A., B.C.Cantab., appointed House Physician to the Paddington Green Children's Hospital. **F. R. Cosgrave**, M.D., B.Ch.Dub., appointed District Medical Officer of the Rochdale Union. **A. W. Woodman Dowding**, M.D., M.S.Dur., M.R.C.P.Edin., M.R.C.S.Eng., appointed Medical District Officer and Public Vaccinator of the St. Columb Union, Cornwall. **G. V. Fletcher**, L.R.C.P., L.R.C.S.Edin., appointed Assistant Medical Officer to the Liverpool Parish Workhouse. **W. Westwood Fyfe**, M.B., C.M.Glasg., appointed Medical Officer for the Postal Staff of the Johnstone Centre, and Medical Officer under the Elementary School Teachers' Superannuation Act. **A. Gregory**, M.R.C.S, L.R.C.P., appointed House Physician to the General Infirmary, Leeds. **T. J. P. Hartigan**, F.R.C.S., appointed Pathologist to the Hospital for Diseases of the Skin, Stamford Street, Blackfriars. **Herbert Hallam**, M.R.C.S., L.R.C.P.Lond., appointed Honorary Anæsthetist to the Sheffield Royal Hospital. **R. Lawford Knaggs**, M.D., M.C., F.R.C.S., appointed Honorary Surgeon to the Leeds General Infirmary. **T. C. Lawson**, M.R.C.S.Eng., appointed Medical Officer for the Southwick Division of the Fareham Union. **C. R. C. Lyster**, M.R.C.S., appointed Medical Officer to the Finsen Light and "X"-Ray Department at the Middlesex Hospital. **Colin McVicar**, M.B., C.M.Edin., appointed Visiting Medical Officer to the Dundee Parish Poorhouse and Hospital. **John Mathewson**, M.B., B.Ch., R.U.I., appointed District Medical Officer of the Bromley Union. **Harold F. Mole**, F.R.C.S.Eng, appointed Surgeon in Charge of the Ear Department of the Bristol Royal Infirmary. **Stanley Moore**, L.R.C.P., L.R.C.S.I, appointed Certifying Factory Surgeon for the Maynooth District of the County of Kildare. **James MacArthur**, L.R.C.P.&P.Edin., L.F.P.S.Glas., appointed House Surgeon to the County Infirmary, Downpatrick, co. Down. **John H. Mackenzie**, M.R.C.S., appointed Health Officer for the Shire of Melton, East Riding, Victoria. **John Barr McLean**, M.B., B.S.Melb., appointed Assistant Medical Superintendent at the Hospital for the Insane, Toowoomba, Queensland. **W. J. Maguire**, B.A., M.B., etc., R.U.I., appointed Medical Officer to the North District of the Royal Irish Constabulary, Belfast. **Henry W. Mason**, L.A.H., appointed Junior House Surgeon to the Jervis Street Hospital, Dublin. **E. J. Miller**, L.S.A.Lond., appointed House Surgeon to the Tynemouth Victoria Jubilee Infirmary, North Shields. **F. Victor Milward**, M.B., B.C.Cantab., F.R.C.S.Eng., appointed Surgical Casualty Officer to the General Hospital, Birmingham. **G. N. Mottram,** M.R.C.S., L.R.C.P.Lond., appointed District Medical Officer of the Williton Union. **D. J. Morgan,** M.B., B.C.Camb., appointed Pathologist of the Cancer Hospital, Fulham Road, S.W. **A. C. Morton,** M.R.C.S.Eng., L.S.A., appointed Medical Officer for the Sixth District and the Workhouse of the Aylsham Union. **Alexander Nicol,** M.D.Aberd., appointed Certifying Factory Surgeon for the Inverurie District of the county of Aberdeen. **A. Stanley Parkinson,** M.B., Ch.B.Vict., appointed Resident Medical Officer of the Worcestershire Sanatorium for Consumption. **Ransom Pickard,** M.S., M.D.Lond., F.R.C.S., appointed Assistant Surgeon to the West of England Eye Infirmary, Exeter. **Sydney Platts,** M.B., B.Ch.Vict., appointed House Surgeon to the General Infirmary, Leeds. **A. L. Rhind,** M.B., Ch.B.Vict., appointed District Medical Officer of the Bingham Union. **C. Rutherford,** L.R.C.P., L.R.C.S.Edin., appointed District Medical Officer of the Hexham Union. **John P. Ryan,** L.R.C.P.&S.I., appointed Senior House Surgeon to Jervis Street Hospital, Dublin. **J. B. O. Richards,** L.R.C.P., L.R.C.S.Edin., appointed District Medical Officer of the Bodmin Union. **W. Stewart,** M.D., appointed District Medical Officer of the Haslingden Union. **T. Scatchard,** L.S.A., appointed House Physician to the General Infirmary, Leeds. **F. J. Stansfield,** M.R.C.S., L.R.C.P., appointed House Surgeon to the General Infirmary, Leeds. **Henry Maurice Tickell,** M.A., M.D., B.C., Cantab., appointed Assistant Physician at Dr. Hettinger's Sanatorium for Open-air Treatment of Lung Diseases, Nordrach, Black Forest, Germany. **H. E Worthington,** M.R.C.S., L.R C.P.Lond., appointed District Medical Officer of the Isle of Thanet Union. **B. Elinor Walters,** M.B., B.S.Lond., appointed Junior Assistant Medical Officer to St. Pancras Infirmary. **A. Garrick Wilson,** M.B.Camb., F.R.C.S.Eng., appointed Senior House Surgeon to the Sheffield Royal Infirmary. **John Wishart,** M.B., Ch.B., B.Sc.Aberd., appointed Deputy Medical Officer of Health to the Bedlingtonshire Urban District Council.

St. Thomas's Hospital.—The following gentlemen have been selected as House Officers from Tuesday, December 2nd, 1902.—House Physicians: **A. D. Hamilton,** M.B.Lond., L.R.C.P., M.R.C.S. (*Extension*); **C. H. Sedgwick,** M.A., M.B., B.C.Cantab. (*Extension*); **W. H. Harwood Yarred,** B.Sc.Lond., L.R.C.P., M.R.C.S.; **A. Mavrogordato,** L.R.C.P., M.R.C.S. Assistant House Physicians: **C. N. Sears,** L.R.C.P., M.R.C.S.; **A. E. Boycott,** M.A., M.B., B.Ch.Oxon, B.Sc.Oxon. House Surgeons (*Extensions*): **W. Hill,** B.A.Cantab., L.R.C.P., M.R.C.S.; **J. Coates,** L.R.C.P., M.R.C.S.; **A. C. Hudson,** M.A., M.B., B.C.Cantab.; **H. Spurrier,** B.A.Cantab., L.R.C.P., M R.C.S. Assistant House Surgeons (*Extensions*): **J. W. Rob,** B.A., M.B., B.C.Cantab.; **T. B. Henderson,** M.A., M.B., B.Ch.Oxon; **J. P. Hedley,** M.A., M.B., B.C.Cantab.; **A. B. Bradford,** M.B., B.S.Durh., L.R.C.P., M.R.C.S. Obstetric House Physicians: (*Senior*) **F. J. Child,** M.A., M.B., B.C.Cantab., L.R.C.P., M.R.C.S.; (*Junior*) **G. A. C. Shipman,** M.A., M.B., B.C.Cantab., L.R.C.P., M.R.C.S. Clinical Assistants: (Throat) **J. E. Adams,** L.R.C.P., M R.C.S. (*Extension*); **S. G. Scott,** M.A., M.B., B.Ch.Oxon (*Extension*). (Skin) **C. Wheen,** B.A.Oxon, L.R.C.P., M.R.C.S. (*Extension*). (Ear) **B. S. Jones,** L.R.C.P., M.R.C.S.; **S. G. Scott,** M.A., M.B., B.Ch.Oxon. (Electrical Department): **O. Hildesheim,** B.A., M.B., B.Ch.Oxon (*Extension*).

**"The Hospital" Institutional Library.**

**"Hospitals and Asylums of the World." 4 Vols., with a Portfolio of Plans, complete £12 12s.**

**"Burdett's Hospitals and Charities, 1902." 5s.**

**"Cottage Hospitals, General, Fever, and Convalescent." 10s. 6d.**

**"Hospital Expenditure: The Commissariat." 2s. 6d.**

**"A Legal Handbook for the Use of Hospital Authorities." 2s. 6d.**

**"The Cottage Hospital Case Book or Register of Patients." 10s. and 12s. 6d.**

**"The Furnishing and Appliances of a Cottage Hospital." 6d.**

**All these are published by the Scientific Press, Ltd., and may be obtained through any bookseller, or direct from the publishers, 28 and 29 Southampton Street, Strand, London, W.C.**

